# *Streptobacillus notomytis* Bacteremia after Exposure to Rat Feces

**DOI:** 10.3201/eid2804.204965

**Published:** 2022-04

**Authors:** Akira Kawashima, Satoshi Kutsuna, Akira Shimomura, Lubna Sato, Honami Ando, Tsutomu Tanikawa, Maki Nagashima, Tohru Miyoshi-Akiyama, Takeshi Inagaki, Norio Ohmagari

**Affiliations:** National Center for Global Health and Medicine, Tokyo, Japan (A. Kawashima, S. Kutsuna, A. Shimomura, L. Sato, H. Ando, M. Nagashima, T. Miyoshi-Akiyama, T. Inagaki, N. Ohmagari);; Yamazaki Professional College of Animal Health Technology, Tokyo (T. Tanikawa)

**Keywords:** *Streptobacillus*, rats, zoonoses, next-generation sequencing, bacteria, *Streptobacillus notomytis*

## Abstract

To determine the source of *Streptobacillus notomytis* bacteremia in a woman in Japan with signs of rat-bite fever, we examined rat feces from her home. After culture and PCR failed to identify the causative organism in the feces, next-generation sequencing detected *Streptobacillus* spp., illustrating this procedure’s value for identifying causative environmental organisms.

Rat-bite fever, characterized by fever, rash, and arthritis, is caused by *Streptobacillus moniliformis* and *Spirillum minus*, which are gram-negative, filamentous, bacilli transmitted by rat bites or ingestion of water or food contaminated with rat feces or urine (*1*). In 2015, *S. notomytis* was reclassified from *S. moniliformis* to the new species (*2*). We reported a case of rat-bite fever in Japan that was caused by *S. notomytis*, identified by 16S rRNA sequencing after blood culture and most likely transmitted by rat feces.

A 70-year-old woman was admitted to the National Center for Global Health and Medicine (Tokyo, Japan), reporting left back pain, lower leg myalgia without arthralgia, and a 3-day fever with abdominal discomfort. At the time of admission, she was fully conscious with the following parameters: temperature 37.9°C, blood pressure 132/68 mm Hg, heart rate 112 beats/min, respiratory rate 24 breaths/min, and oxygen saturation 98%. She reported that rats often entered her apartment and that she had noticed the smell of urine over the previous 2 weeks. She had no rash or bite wounds, and her conjunctiva appeared normal. Chest radiographs showed no signs of pneumonia.

Laboratory tests revealed leukocytosis (9.0 × 10^8^ cells/L) and elevated C-reactive protein concentration (16.9 mg/dL). Urinalysis revealed hematuria but no other signs of pyelonephritis. Contrast-enhanced computed tomography of the trunk revealed no kidney abnormalities.

Because hematuria led us to suspect pyelonephritis, we initiated ceftriaxone treatment (2 g/d), after which, the patient’s fever rapidly resolved. On day 2, diffuse purpura appeared on both of her legs but rapidly resolved. Blood culture revealed gram-negative bacilli (Figure, panel A). On the basis of her history of rat contact and blood culture results, we provisionally diagnosed rat-bite fever but continued administering ceftriaxone.

**Figure:**
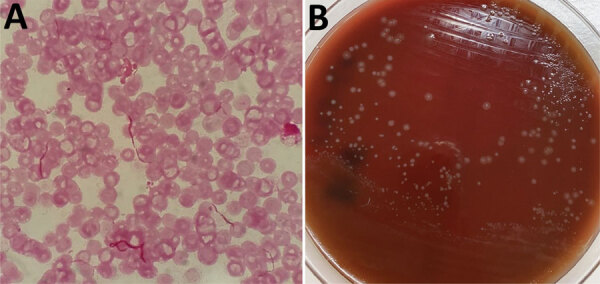
Results of testing for the causative agent of rat-bite fever in a 70-year-old woman in Japan. A) Gram-stained blood smear showing chain-shaped gram-negative bacilli; original magnification ×1,000. B) Small, smooth colonies in culture of healthy human serum (provided by one of the authors of this article) on 5% sheep blood agar. Further testing identified the bacilli as *Streptobacillus notomytis*.

Bacterial culture of the patient’s blood yielded small, smooth colonies on 5% sheep blood agar and 5% horse blood agar (both Nissui Pharmaceutical, https://www.nissui-pharm.co.jp) with addition of healthy serum (provided by one of the authors of this article) and incubation at 35°C under 7% carbon dioxide for 48 h (Figure, panel B). We evaluated the isolate by using matrix-assisted laser desorption/ionization time-of-flight mass spectrometry with the Bruker MALDI BioTyper software version 6.903 library database (Bruker Daltonik GmbH, https://www.bruker.com). *S. moniliformis* was a candidate, but its score of 1.49 was considered too low. We determined antimicrobial susceptibility patterns by using broth microdilution. The MIC of oxacillin was <0.12 μg/mL; ampicillin, <0.12 μg/mL; cefazolin, <0.5 μg/mL; imipenem, 0.25 μg/mL; vancomycin, <0.5 μg/mL; clindamycin, <0.06 μg/mL; and levofloxacin, <0.5 μg/mL. To further characterize the isolate, we performed 16S rRNA gene sequencing by using a universal primer pair: 5F (5′-TTGGAGAGTTTGATCCTGGCTC-3′ and 1485B (5′-TACGGTTACCTTGTTACGAC-3′). The sequence showed 100% identity (1,465/1,465 bp) with *S. notomytis* AHL_370–1 (GenBank accession no. KR001919). Therefore, on day 6, we changed treatment to intravenous ampicillin (2 g 4×/d). On day 7, treatment was changed to oral amoxicillin (500 mg 3×/d), and the patient was discharged. She completed a 14-day course of ceftriaxone, ampicillin, and amoxicillin and showed no further signs of bacteremia.

After the patient was discharged, we visited her home and collected rat feces samples. We saw a rat in her home but were unable to capture it. The rat feces were dry, and the target bacteria could not be cultured. Because the causative organism could not be identified by wide-range 16S rRNA PCR due to the large number of species of bacteria, we performed next-generation sequencing of DNA extracts as described previously (*3*), prepared library NEBNext Ultra II DNA Library Prep Kit (New England BioLabs, https://www.neb.com) according to the manufacturer’s instructions, and analyzed sequence data by using MiSeq (Illumina, https://www.illumina.com). Results confirmed the presence of *Streptobacillus* spp. in the rat feces. We used the *S. notomytis* genome as a reference sequence (GenBank accession no. GCA_00161275.1) for comparison of sequence reads. We performed mapping with a 99% identity setting. Of the 844,070,6 genes read, 177 were mapped and possibly belong to *S. notomytis*.

Three cases of *S. notomytis* infection in humans have been reported; the pathogen was detected in the blood, skin pustules, and synovial fluid (*4*–*6*). In the case we report, *S. notomytis* was identified in the blood by using 16S rRNA sequencing. *S. moniliformis* and *S. notomytis* may also cause Haverhill fever, which is epidemic arthritic erythema transmitted by inhalation or ingestion of food and drink contaminated with rat feces and urine (*7*–*10*). Because the patient reported here had no history of rat bites, the most likely mode of transmission was aerosol inhalation of dried rat feces or water contamination at the patient’s home.

*S. moniliformis* infection is usually treated with penicillin, cephalosporins, or tetracyclines (*1*). *S. notomytis* is also reportedly sensitive to these drugs, but its sensitivity to amoxicillin is reportedly intermediate (*4*,*5*). For this patient, ceftriaxone and ampicillin were prescribed. In summary, after conventional methods (culture and PCR) failed to identify the causative organism for this patient’s infection, we detected *Streptobacillus* spp. in rat feces by using next-generation sequencing, thereby illustrating the value of this procedure for identifying causative organisms in the environment.
